# A Transcriptomic Pipeline Adapted for Genomic Sequence Discovery of Germline-Restricted Sequence in Zebra Finch, *Taeniopygia guttata*

**DOI:** 10.1093/gbe/evab088

**Published:** 2021-04-26

**Authors:** Kathryn C Asalone, Ajuni K Takkar, Colin J Saldanha, John R Bracht

**Affiliations:** 1 Department of Biology, American University, Washington, District of Columbia, USA; 2 Department of Neuroscience, American University, Washington, District of Columbia, USA; 3 Center for Neuroscience and Behavior, American University, Washington, District of Columbia, USA

**Keywords:** germline-restricted chromosome, GRC, zebra finch, comparative coverage analysis, FPKM, sequence discovery, intronless transcriptomics

## Abstract

Songbirds have an unusual genomic element which is only found in their germline cells, known as the germline-restricted chromosome (GRC). Because germ cells contain both GRC and non-GRC (or A-chromosome) sequences, confidently identifying the GRC-derived elements from genome assemblies has proven difficult. Here, we introduce a new application of a transcriptomic method for GRC sequence identification. By adapting the Stringtie/Ballgown pipeline to use somatic and germline DNA reads, we find that the ratio of fragments per kilobase per million mapped reads can be used to confidently assign contigs to the GRC. Using this comparative coverage analysis, we successfully identify 733 contigs as high confidence GRC sequences (720 newly identified in this study) and 51 contigs which were validated using quantitative polymerase chain reaction. We also identified two new GRC genes, one hypothetical protein and one gene encoding an RNase H-like domain, and placed 16 previously identified but unplaced genes onto their host contigs. With the current focus on sequencing GRCs from different songbirds, our work adds to the genomic toolkit to identify GRC elements, and we provide a detailed protocol and GitHub repository at https://github.com/brachtlab/Comparative_Coverage_Analysis (last accessed May 12, 2021).

Significance

The presence of the germline-restricted chromosome (GRC) in the zebra finch, and various other songbirds is widely known, however, an effective process for discovering sequences and genes has yet to be described. This study introduces a new application of an existing transcriptomic method and this pipeline, termed comparative coverage analysis, was used to identify 733 contiguous sequences in the zebra finch genome (720 new). This method may be utilized across songbird species in order to discover GRC sequences and genes.

##  

The zebra finch (*Taeniopygia guttata*) is a well-established model organism for studies in neuroendocrinology and vocal learning, and also undergoes developmentally programmed genome rearrangement (Pigozzi and Solari 1998, 2005). This characteristic is unusual in vertebrates, making the zebra finch a member of a select group of amniotes with dramatically labile genomes (Wang and Davis 2014). The genome rearrangement in the finch occurs through the developmentally dynamic elimination of a distinctive germline-restricted chromosome (GRC). Most famously, in males, the GRC is present in spermatogonia but is typically eliminated during the development of mature sperm, though recent work suggests that this is imperfect, leading to nonnegligible levels of paternal GRC inheritance (Pei et al. 2021). The GRC is retained in mature ova of the female, and in both sexes it is apparently expelled from all somatic cells early on in embryonic development (Pigozzi and Solari 2005). This leaves the inheritance of the GRC primarily to the females, where transmission occurs through the oocyte (Pigozzi and Solari 2005).

Over the past few years the literature on the GRC in songbirds has been expanding (Biederman et al. 2018; Kinsella et al.^ 2019^; Torgasheva et al.^ 2019^; Malinovskaya et al. 2020). Using subtractive genomics, our lab discovered the first protein-coding GRC gene in the zebra finch, the GRC-linked α-Soluble NSF Attachment Protein (GRC α-SNAP, also called *NAPAG*) (Biederman et al. 2018). This discovery included the identification of a paralogous A-chromosome copy (or somatic paralog: somatolog) which, following α-SNAP naming conventions, is the *NAPA* gene. The existence of somatologs in the germline presents a computational challenge for subtractive methods to identify the GRC, as A-chromosome reads can errantly map to the GRC element and vice versa.

Highlighting the pervasive nature of this problem, Kinsella et al. sequenced the entirety of the GRC and identified 92 paralogous segments constituting the complete GRC, showing that it is largely patched together from copies of sequences existing on the A-chromosome (Kinsella et al. 2019). In addition to the 92 paralogous segments, this pioneering study also yielded 245 GRC genes (115 high confidence) and 36 high-confidence GRC contigs housing 21 genes; however the rest of the identified GRC genes (224) remain unplaced and many more GRC contigs remain to be identified from the germline assembly (Kinsella et al. 2019).

To date, methods for identifying GRC include somatic-mapping coverage analysis and snp analysis. Somatic-mapping coverage analysis involves mapping the germline-derived sequencing reads onto the somatic genome assembly (Kinsella et al. 2019). This method was used to identify 92 paralogous regions on the A-chromosomes which exhibit a 4-fold increase from expected in germline-read coverage (Kinsella et al. 2019). Similarly, mapping germline reads to somatic reference enabled the identification of 245 genes based in single-nucleotide polymorphisms (Kinsella et al. 2019). Since these methods rely on an existing target on the A-chromosome to identify GRC elements, unique GRC content cannot be identified as it would not present a paralogous A-chromosome target for read mapping. In another drawback, specific GRC contigs are not isolated and instead the GRC sequence must be imputed from the mapping back onto the germline assembly, lowering the overall sensitivity of the method.

We speculated that by mapping somatic and germline reads directly to the germline assembly and analyzing the resultant differential coverage with transcriptomic software, such as HISAT–Stringtie–Ballgown (Pertea et al. 2016), we could identify GRC elements in an unbiased manner and with greater sensitivity. Because Ballgown is built to directly measure differential gene expression across two or more experimental conditions (Pertea et al. 2016), it is well suited to comparing germline versus somatic coverage of the whole testis assembly, which includes both GRC and A chromosome sequences (fig. 1*A* and *B*). Not only does HISAT–Stringtie–Ballgown provide robust statistical discrimination, but by using differential fragments per kilobase of transcript per million mapped reads (FPKM) we also can normalize for sequencing library depth across replicates (fig. 1*A* and *B*). To accomplish this we tricked the pipeline to tolerate a lack of introns by manually adjusting the input and output files for Stringtie. In this manuscript we show this method (see method workflow, fig. 1*C*), succeeds in identifying many previously undetected GRC elements. We provide a detailed protocol and all associated scripts in supplementary methods, Supplementary Material online and at https://github.com/brachtlab/Comparative_Coverage_Analysis (last accessed May 12, 2021).

**Fig. 1.**
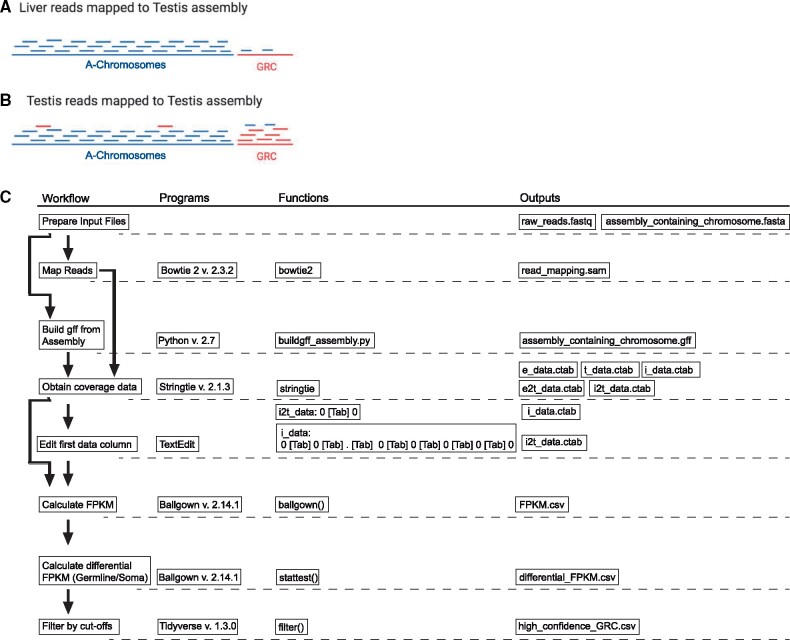
(*A*) Visualization of expected read coverage of liver raw reads mapped onto a testis assembly. (*B*) Visualization of expected read coverage of testis raw reads mapped onto a testis assembly. Blue represents A-chromosome sequences/reads, whereas red represents germline-restricted sequences/reads. (*C*) Schematic representation of methods. Outputs from the previous step feed into the next step as the input. [Tab] represents a tab in the text as entered in TextEdit software.

Our method specifically accounts for mismapping due to paralogy, helping to avoid pitfalls of the genomic subtraction method (Biederman et al. 2018; Asalone et al.^ 2019^). For example, if somatic (liver or leg) raw reads were mapped onto a testis assembly, the reads would mainly map on to the A-chromosomes with a few mismapping on the GRC-derived sequence (fig. 1*A*) (these mismapping are expected since all of the known GRC sequences have A-chromosome counterparts). We further theorize that in the case of germline (testis) raw reads mapped on to a testis assembly, the reads should distribute more evenly across the entire genome, with high coverage of true GRC sequences and with a few reads mismapping to the A-chromosomes because of paralogy (fig. 1*B*). By taking a ratio of germline to somatic coverage for each contig, we normalize for both the mismapping of reads and for variation in copy number of both A-chromosome and GRC sequences. To obtain statistical significance, we combined data from multiple individual birds as technical replicates, deriving the average and standard deviation for each contig.

We applied this method, which we call comparative coverage analysis, to Kinsella et al.'s publicly available testis genome assemblies (Testis Assembly, Kinsella et al. 2019), as well as matching somatic and testis genomic reads from four birds: three from Kinsella et al. (2019), and one from our stock (Biederman et al. 2018; Raw Reads, Kinsella et al. 2019); thus we had *n* = 4 total somatic and *n* = 4 corresponding germline read sets. All input data were adaptor-trimmed but otherwise raw; we did not use pair data sets, just the forward read files in fastq format. For a mapping target we used the phased P7359_106 testis assembly (Kinsella et al. 2019) and generated a volcano plot of the results (fig. 2*A*); we have also tested the unphased assembly, with largely similar results (supplementary fig. S3, Supplementary Material online).

**Fig. 2.**
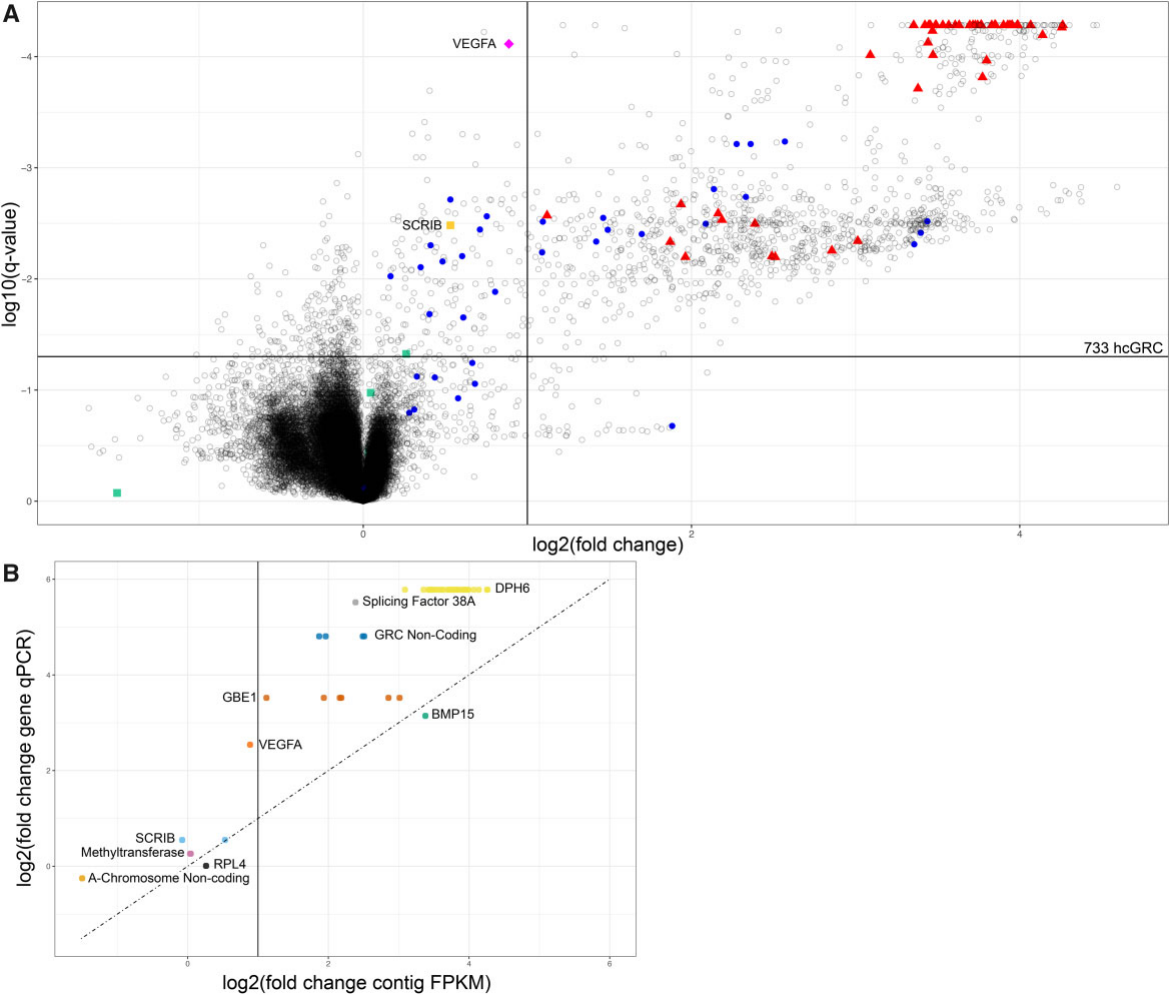
(*A*) Volcano plot of fragments per kilobase per million reads mapped (FPKM) fold change comparing testis (*n* = 4) and somatic (*n* = 4) data sets. The vertical line represents a fold change of 2, the horizontal line represents a *q*-value of 0.05. Unknown contigs are represented by empty, black, opaque (alpha = 0.25) circles; contigs validated in this study by qPCR are represented by red triangles; 36 contigs identified as GRC from Kinsella et al. are represented by blue circles. The contig identified as GRC from Kinsella et al. and validated in this study (vascular endothelial growth factor A, *VEGFA*) is a pink diamond whereas the scribble planar cell polarity protein, *SCRIB* contig, not predicted to be GRC in our analysis, is a yellow square; negative control contigs are represented by green squares. (*B*) Comparison of fold change of gene in genomic qPCR (testis/liver DNA) versus fold change of contig FPKM derived by comparative coverage analysis. The dotted line represents the 1:1 line. The solid horizontal line represents a 2-fold change in FPKM. Note, for most qPCR targets there are multiple contigs yielding FPKM values because of the repetitive nature of the GRC. Diphthine-ammonia ligase (*DPH6*) is represented in yellow, splicing factor 38A in gray, GRC noncoding sequence in navy blue, 1,4-alpha-glucan branching enzyme (*GBE1*) in red, bone morphogenetic protein 15 (*BMP15*) in green, vascular endothelial growth factor A (*VEGFA*) in orange, scribble planar cell polarity protein (*SCRIB*) in light blue, methyltransferase in pink, ribosomal protein L4 in black and A-chromosome noncoding sequence in light orange.

Mapping of genomic reads does not require splice-awareness, so in place of HISAT, we deployed Bowtie 2 v. 2.3.2 (Langmead and Salzberg 2012), because of its efficiency and also because it reports a single best alignment for each read (Langmead and Salzberg 2012). After Bowtie2 mapping, the Stringtie–Ballgown pipeline (Pertea et al. 2016) was used to generate FPKM for each contig from somatic or germline reads, and germline-soma differential *q*-values (corrected for false discovery rate) were obtained with the stattest() function. Both Bowtie2 and stattest() were run using default parameters. To perform the analysis with Stringtie, we built a gff file in which each contig is demarcated as a single transcript (using buildgff_assembly.py). We also manually adjusted the stringtie output files because a lack of introns otherwise caused error messages (see supplementary methods, Supplementary Material online and https://github.com/brachtlab/Comparative_Coverage_Analysis, last accessed May 12, 2021 for details).

A volcano plot of differential coverage shows a clear separation between the A-chromosome contigs and possible germline-restricted contigs (fig. 2*A*). We set a 2-fold germline-to-soma fold-change, *q*-value of below 0.05, and length threshold of >2,000 bp to demarcate high confidence GRC, hcGRC, contigs (*n* = 733 contigs) (fig. 2*A*, supplementary table S1, Supplementary Material online). By manually examining these contigs, we identified at least two novel GRC genes (table 1). We also placed 19 of the previously published genes (Kinsella et al. 2019) onto contigs from the hcGRC list (supplementary table S1, Supplementary Material online); however, 23 of the previously published contigs did not show strong germline enrichment by our coverage analysis (fig. 2*A*) suggesting they either are not multi-copy or are highly similar to an A-chromosome paralog and thus are not identified by our method (we discuss more limitations of our method below). Although previous work placed 21 genes onto GRC contigs (Kinsella et al. 2019), only 3 of these overlap with the 19 we placed, suggesting that 18 of the 21 previously placed genes are encoded by contigs of low copy number and high similarity to A-chromosome paralogs that are not well suited to our method. We selected six putative hcGRC for quantitative polymerase chain reaction (qPCR) validation, representing various degrees of coverage enrichment, including one on the borderline of the 2-fold cutoff (but predicted by Kinsella et al.) and four additional negative controls (fig. 2*A*).

**Table:** **Table 1**. Newly Described GRC Genes

Gene	Function	Contig ID	FPKM Fold Change	Coordinate Start	Coordinate Stop
Hypothetical protein	N/A	739	10.93	27,383	25,784
		740	5.55	27,088	25,486
		979	10.92	3,688	3,539
		980	15.89	3,691	3,542
		1600	9.00	506	16,387
		1851	7.00	13,020	10,895
		2223	9.37	319	8,595
		2224	5.73	319	8,610
		151423	3.47	273	1
		168518	11.67	2,943	441

LOC105760826	HMMER identifies an RNase H-like domain found in reverse transcriptase (PF17919) (Finn, Clements, and Eddy 2011).	163675	3.90	2,057	213

By qPCR we found that six regions have a significantly greater detection in testis versus liver DNA (supplementary fig. S1, Supplementary Material online). These six sequences were found on 51 contigs in the assembly, which are now considered validated hcGRC. These contigs largely reside within the specified GRC cut-offs identified in this study (fig. 2*A* and *B*). The borderline case (*VEGFA*), originally identified by snp analysis (Kinsella et al. 2019), was confirmed by qPCR (supplementary fig. S1, Supplementary Material online and fig. 2*A* and *B*), showing the 2-fold cutoff for the volcano plot is conservative. None of the negative control contigs were validated as GRC by qPCR (supplementary fig. S2, Supplementary Material online and fig. 2*A* and *B*).

We investigated how many replicate birds should be sequenced for our method. Unsurprisingly, more replicates are better: when only two replicates (*n* = 2 germline and *n* = 2 corresponding somatic data sets from the same two birds) were used, no contigs were identified as statistically significant; when three replicates were used, 506 contigs were identified whereas four replicates allowed the identification of 733 contigs (at 2-fold enrichment and *q*-value < 0.05). Thus, we conclude that at least three replicate animals (with one germline and one somatic data set per animal) are necessary, with increased sensitivity at higher replicate numbers. This finding concurs with previous studies showing at minimum three replicates are needed for RNA-seq experiments to yield statistically robust results (Schurch et al. 2016; Lamarre et al. 2018). We also investigated whether phasing the assembly makes a difference. When running the comparative coverage analysis on the unphased assembly (Kinsella et al. 2019), 591 contigs are identified as hcGRC (supplementary fig. S3, Supplementary Material online). Using reciprocal BLAST analysis, we compared the contigs identified as hcGRC from phased versus the unphased assemblies. Consistent with the lower contig number (unphased, 40,179 vs. 41,343 for phased) and greater contiguity (unphased N50 = 17.6 Mb vs. 7.32 Mb for phased) of the unphased assembly, the 733 phased hcGRC contigs map onto 543 unphased contigs as reciprocal BLAST matches (RBMs). Reassuringly, however, these RBMs largely map to hcGRC. Of the 543 RBMs, 540 (99%) are included in the 591 unphased hcGRC contigs, with 51 contigs (9%) uniquely identified in the unphased assembly. Thus, evaluating either phased or unphased assemblies identify largely the same contigs, suggesting our method is fairly robust to the underlying genomic assembly method. We chose to present the phased assembly results in figure 2*A* because we can draw direct comparisons to Kinsella et al.’s findings.

Our method has limitations. Single-copy elements are expected to be more suitably detected using snp-based methods as utilized by Kinsella et al. More generally, we expect there to be three distinct categories of sequences present on the GRC, with our method effectively identifying the first two: 1) high-copy, 2) low-copy divergent, and 3) low-copy similar. The first category consists of GRC sequences that are multicopy, predicted to have significant fold change driven by a large FPKM in the germline reads. In these cases, the numerator (germline coverage) drives a high fold-change in the germline-to-somatic mapping ratio.

The second category consists of sequences that are low or single copy yet relatively divergent from all A-chromosome paralogous sequences. Examples of these sequences include *NAPAG* (Biederman et al. 2018) and *BMP15* which, by qPCR, seems to be single copy in germline (detection relative to actin ∼1, supplementary fig. S1*B*, Supplementary Material online) while being divergent enough that the A-chromosome paralog does not amplify well; likewise somatic reads will map only sparsely onto these elements. For these elements, a high germline-to-somatic mapping level will be driven by a small FPKM from the somatic reads, or the denominator of the fold-change ratio.

The final category, single-copy but nearly identical to A-chromosome paralogs, are practically indistinguishable by sequence from their paralogous copies, so a 2-fold enrichment will not be met assuming a haploid testis GRC. In this case, two A-chromosome alleles plus one GRC allele will give a ratio of 3:2 for germline versus somatic mapping ratio. As an example of this, Contig 164558 (with Scribble Planar Cell Polarity Protein, *SCRIB*) was identified as GRC in Kinsella et al., however, both the FPKM fold-change and qPCR showed a mild 1.4-fold enrichment consistent with the 3:2 ratio expected if the GRC provides only one paralog. Another example of this is contig 1639 (carries vascular endothelial growth factor A, *VEGFA*), which was identified as GRC by Kinsella et al., however, did not meet the 2-fold enrichment threshold by FPKM (pink diamond, fig. 2*A*). This challenging class of sequence is best identified by snp analysis because no other method is capable of identifying GRC genes; however, from our analysis, using the fold change of *SCRIB* (1.45) as the cutoff as well as a *q*-value of 0.05, we estimate there are approximately 71 such contigs, six of which are among the 36 identified in Kinsella et al.

There is significant complementary value in snp-based and comparative coverage analysis methods. Although Kinsella et al. identified 36 contigs, our method identifies only 13 of those, but adds 720 more. In the future, a way to combine both snp-based and comparative coverage-based analysis may be the best way to comprehensively identify GRC elements from a germline assembly. Here, we show that our method for isolating multi-copy GRC genes and sequences is robust to the paralogous nature of the zebra finch GRC genome, which can stymie subtractive methods. We have experimentally validated the parameter space, and our stringent thresholds yield a high number (*n* = 733) of high-confidence GRC contigs, 720 of which are newly confirmed in our study. We also placed 16 previously unplaced genes from Kinsella et al. onto these 733 contigs, helping to fill out our genomic understanding of the GRC.

We estimate that the total length of hcGRC that we have identified, using germline coverage of each contig to compensate for collapse of multicopy sequences, is 55.3 million bp (see supplementary methods, Supplementary Material online). The hcGRC length of 55.3 million bp accounts for 46% of the GRC (assuming the total GRC length is 120 million bp, which is a conservative estimate based on length of chromosome 1 and the observed GRC length in karyotypes). This approximation for the length of repetitive and low-copy divergent GRC categories (categories 1 and 2 together) indicated that significant amounts of sequence remain undiscovered, which we expect to derive largely from category 3 (low-copy number and very similar to their A-chromosome paralogs).

Our work shows that computational methods that initially were designed for one purpose (i.e., differential gene expression) may be co-opted to new uses. Because we adapted the Stringtie–Ballgown pipeline to use intronless “transcripts” (representing one entire contig as a transcript), our modifications of the pipeline may facilitate other research including prokaryotic or mitochondrial gene expression where introns are lacking. This method may also be effective in identifying variable genomic elements, such as B- or Y-chromosomes. As long as cells with and without the chromosome in question can be sequenced and an assembly containing the chromosome in question can be produced, our method should be applicable. For example, when evaluating an organism using the standard XY sex determination system, to identify the Y-chromosome male and female siblings may be sequenced and coverages compared. The method may also be able to distinguish X-chromosome elements by female-coverage enhancement relative to male coverage. In the near future, we aim to apply this method, and the complementary snp analysis, to more songbirds to help address open questions about where the GRC arose, what GRC elements are common across species, and ultimately, what is the biological function of this remarkable genomic component.

## Supplementary Material

evab088_Supplementary_Data Supplementary data are available at *Genome Biology and Evolution* online.

## Data Availability

Custom python scripts can be found in the Github repository, Comparative_Coverage_Analysis (https://github.com/brachtlab/Comparative_Coverage_Analysis, last accessed May 12, 2021). Data generated in this study have been deposited into the Sequence Read Archive for DNA and RNA sequencing data (BioProject accession number PRJNA698222 [https://www.ncbi.nlm.nih.gov/sra/PRJNA698222]).
