# Supplementary material for: A Transcriptomic Pipeline Adapted for Genomic Sequence Discovery of Germline-Restricted Sequence in Zebra Finch, Taeniopygia guttata
Source: Genome Biol Evol. 2021 Apr 26;13(6):evab088. doi: 10.1093/gbe/evab088 (PMC8245190; doi:10.1093/gbe/evab088)
Supplement: evab088_Supplementary_Data — Supplementary data are available at Genome Biology and Evolution online. [file evab088_supplementary_data.zip › Supplemental Methods.docx]

Supplemental Methods

*Comparative Coverage Analysis*

We utilized the publicly available testis assembly, as well as matching somatic and testis genomic reads from four birds: three from [(Kinsella et al. 2019)](https://paperpile.com/c/qiUfmY/v6X2), and one from our stock [(Biederman et al. 2018)](https://paperpile.com/c/qiUfmY/mKXDU); thus we had n=4 total somatic and n=4 corresponding germline read sets. All input data were adaptor-trimmed but otherwise raw; we did not use pair datasets, just the forward read file in fastq format. However, forward and reverse reads can be used if desired. For a mapping target we used the phased P7359_106 testis assembly [(Kinsella et al. 2019)](https://paperpile.com/c/qiUfmY/v6X2). In order to align the raw reads to the testis assembly, Bowtie 2 v. 2.3.2 [(Langmead and Salzberg 2013)](https://paperpile.com/c/qiUfmY/gjyf8) was run on default settings, outputting a sam alignment file for each read set. The sam files were then converted to bam files, and coordinate sorted using Samtools v. 1.9 [(Li and Durbin 2009)](https://paperpile.com/c/qiUfmY/29rB4).

Once the read mapping files were coordinate sorted, each bam file was run through Stringtie v. 2.1.3b. [(Pertea et al. 2016)](https://paperpile.com/c/qiUfmY/k4wiV). Stringtie was run using settings -t, to disable trimming, -e, to only output assembled transcripts that match reference transcripts, -b, to create outputs that are processed using Ballgown, and -G, to input a reference annotation file. The reference annotation file was created from the testis assembly using custom python script, buildgff_assembly.py. The Ballgown package v. 2.14.1 [(Pertea et al. 2016)](https://paperpile.com/c/qiUfmY/k4wiV) expects data on both exons and introns, however, since we calculated FPKM for whole genomes, where each contig was classified as a transcript, no intron data was present. The Stringtie output files i_data.ctab and i2t.ctab were empty, therefore, for each run, these files required modifications to tell the program that no introns were present. For i_data.ctab a 0 was added to the first row of each column, except the strand column in which a `.` was input, in the i2t.ctab file a 0 was added to the first row in each column. Each read set output was then loaded into RStudio [(Team 2015)](https://paperpile.com/c/qiUfmY/rC1yi) where Ballgown was used to identify FPKM. The stattest() function, a part of the Ballgown package, was then used to calculate the fold change of FPKM for each contig from somatic reads to germline reads, data was plotted using ggplot2 v. 3.3.2 [(Wickham 2011)](https://paperpile.com/c/qiUfmY/6DOv6).

*DNA/RNA Extraction*

Adult zebra finches (Taeniopygia guttata) were obtained from a commercial breeder and housed in groups (15-25 per cage) in samesex aviaries. Subjects were rapidly decapitated and tissue was removed and flash frozen on dry ice. Samples were then weighed and stored at 80 degrees until further processing. Tissues were homogenized in 500 uL 100 mM Phosphate Buffer pH 7.4. In order to extract DNA from 100 uL of homogenized tissue from two testis and two ovary samples, the Quick DNA/RNA MiniPrep Plus kit (Zymo Research Cat D7003) was used. The homogenized tissue was mixed with 100 uL of DNA/RNA Shield buffer, 20 uL of PK Digestion Buffer, and 10 uL of Proteinase K. The mixture was vortexed and incubated at 55℃ for 30 min. After the incubation period, the mixture was vortexed for 2 min and centrifuged for 2 min before transferring the supernatant and adding 200 uL of lysis buffer. The mixture was transferred to yellow (for DNA) columns according to the manufacturer instructions. The concentration of DNA was analyzed using Invitrogen Qubit assays.

*Next-generation sequencing*

DNA was sequenced at the Genomics Core Facility at Icahn School of Medicine (NY, NY). The libraries were constructed from the extracted liver and testis DNA samples with insert sizes of 155bp and sequenced in paired-mode on a HiSeq2500. Read lengths were 100bp. Raw forward reads were adaptor trimmed with Trimmomatic v. 0.33 prior to performing mapping and coverage analysis. Testis reads were sequenced in 3 runs giving 3 files.

*qPCR Validation*

To identify which contigs have potential genes, 245 gene sequences, that were identified from Kinsella *et al.* [(Kinsella et al. 2019)](https://paperpile.com/c/qiUfmY/v6X2), were used as the blastn query to the hcGRC contigs. All qPCR reactions (Table S2) were run using a 1-stage cycle with 95℃ for 10 min initial melt, then 40 cycles of 95℃ for 30 s, 55℃ for 1 min and measurement of DNA concentration. All signals were measured relative to actin by ΔCt. The average and standard deviation of $2^{-(geneCt-\beta-actinCt)}$ were calculated for all cases. Statistical significance was measured by Student’s 2-tailed t-test.

*Comparison of Phased to Unphased Assembly*

To test if the phased assembly works best with our pipeline, we utilized the comparative coverage analysis using the unphased assembly. A reciprocal blast match was performed to directly compare results of our method when raw reads were mapping to the phased assembly versus the unphased assembly. To do this, a blastn [(Altschul et al. 1990)](https://paperpile.com/c/qiUfmY/gqgu) search, where the phased assembly was used as the query, the unphased assembly as the database and flags -outfmt 6 and -max_target_seqs 1 were used to create a tabular output and only report the top hit, respectively. The same procedure was followed where the unphased assembly was the query while the phased assembly was the database. The two blast outputs were then run through the custom python script, RBM.py, which outputs a list of the reciprocal blast matches (RBM). RBM are determined when the same query and database contigs match despite which assembly is used for each input. For example, when the unphased is the query, contig 0 matches to the phased contig 36, when the phased is the query, contig 36 matches to unphased contig 0. These are considered reciprocal pairs and the RBM.py will output 0 and 36 as being reciprocal blast matches along with all other RBM across the two assemblies. This list of RBM was used to compare the contigs identified as high confidence GRC from the unphased assembly to the phased hcGRC. To do this, a custom python script, get_names_RBM.py, takes the list of contig IDs from the phased hcGRC (n=733) and pulls the unphased contig ID from the RBM output. This list of unphased contigs IDs could then be compared to those present in the hcGRC unphased contig list.

*Length Estimation*

To approximate the length of the hcGRC, we first estimated what germline FPKM (raw FPKM only of testis reads, not the ratio) corresponds to the single-copy elements. This was performed by generating a histogram which showed a distinct peak at an FPKM of most sequences at 0.8, suggesting that this value corresponds to single-copy elements. This was validated by identifying the FPKM of three contigs on which known single-copy genes (which we confirmed by blast analysis); actin-related protein 2/3 complex subunit 2 (ARPC2, NM_001245266.1), glycine receptor subunit alpha-4 (LOC100221380, XM_002190092.4), apoptotic protease-activating factor 1 (APAF1, XM_030272315.2), lie (FPKM = 0.809, 0.783, 0.790 respectively). An approximate copy number for each contig was evaluated by dividing the contig’s FPKM by our single-copy value of 0.8. To further confirm copy number calculations, we estimated the combined copy number of DPH6 contigs, which we found by qPCR to have 582 copies in the germline (Figure S2); our FPKM approximated 575 copies. Therefore, our method agrees very well with known contigs. Estimated length of the hcGRC was determined by summing each contig’s copy number multiplied by its length.

**Supplemental Fig 1.** Genomic DNA qPCR analysis confirming GRC A. Splicing Factor 38A, B. Bone Morphogenetic Protein 15, C. Vascular Endothelial Growth Factor A, D. Diphthine--Ammonia Ligase, E. 1,4-alpha-glucan branching enzyme 1, and a F. Non-Coding Sequence are detected significantly more in testis tissue rather than liver tissue. Error bars represent the standard deviation, t-test identified statistical significance (* = p < 0.05, ** = p < 0.01, *** = p < 0.001).

**Supplemental Fig 2.** Genomic DNA qPCR analysis confirming non-GRC sequences. A. Scribble Planar Cell Polarity Protein B. Methyltransferase C. A-Chromosome Non-Coding D. Ribosomal Protein L4. Sequences were not detected significantly more in either testis or liver tissue. Error bars represent the standard deviation, t-test identified statistical significance.

**Supplemental Fig 3.** Volcano plot of fragments per kilobase per million reads mapped (FPKM) fold change from unphased assembly, comparing testis (n=4) and somatic (n=4) datasets. The vertical line represents a fold change of 2, the horizontal line represents a q-value of 0.05. Unknown contigs are represented by empty, black, opaque (alpha = 0.25) circles; contigs validated in this study by qPCR are represented by red triangles; RBM contigs identified as GRC from Kinsella *et al*. are represented by blue circles. The contig identified as GRC from Kinsella *et al*. and validated in this study (vascular endothelial growth factor A, *VEGFA*) is a pink diamond while the scribble planar cell polarity protein, *SCRIB* contig, not predicted to be GRC in our analysis, is a yellow square; negative control contigs are represented by green squares.

**Supplemental Table 1.** List of 733 contigs, validated gene, proposed category (1 = high-copy, 2 = low-copy divergent), length, q-value, fold change, blastn match to Kinsella *et al*. 245 genes and coordinates.

**Supplemental Table 2.** Primers used for qPCR

References

[Altschul, S. F., W. Gish, W. Miller, E. W. Myers, and D. J. Lipman. 1990. “Basic Local Alignment Search Tool.” *Journal of Molecular Biology* 215 (3): 403–10.](http://paperpile.com/b/qiUfmY/gqgu)

[Biederman, Michelle K., Megan M. Nelson, Kathryn C. Asalone, Alyssa L. Pedersen, Colin J. Saldanha, and John R. Bracht. 2018. “Discovery of the First Germline-Restricted Gene by Subtractive Transcriptomic Analysis in the Zebra Finch, Taeniopygia Guttata.” *Current Biology: CB* 28 (10): 1620–27.e5.](http://paperpile.com/b/qiUfmY/mKXDU)

[Kinsella, Cormac M., Francisco J. Ruiz-Ruano, Anne-Marie Dion-Côté, Alexander J. Charles, Toni I. Gossmann, Josefa Cabrero, Dennis Kappei, et al. 2019. “Programmed DNA Elimination of Germline Development Genes in Songbirds.” *Nature Communications* 10 (1): 5468.](http://paperpile.com/b/qiUfmY/v6X2)

[Langmead, B., and S. L. Salzberg. 2013. “Langmead. 2013. Bowtie2.” *Nature Methods* 9: 357–59.](http://paperpile.com/b/qiUfmY/gjyf8)

[Li, Heng, and Richard Durbin. 2009. “Fast and Accurate Short Read Alignment with Burrows–Wheeler Transform.” *Bioinformatics*  25 (14): 1754–60.](http://paperpile.com/b/qiUfmY/29rB4)

[Pertea, Mihaela, Daehwan Kim, Geo M. Pertea, Jeffrey T. Leek, and Steven L. Salzberg. 2016. “Transcript-Level Expression Analysis of RNA-Seq Experiments with HISAT, StringTie and Ballgown.” *Nature Protocols* 11 (9): 1650–67.](http://paperpile.com/b/qiUfmY/k4wiV)

Raw reads. Kinsella et al. 2019. [Programmed DNA Elimination of Germline Development Genes in Songbirds](http://paperpile.com/b/ggxh4E/FHid). Sequence Read Archive. PRJNA552984. <https://www.ncbi.nlm.nih.gov/bioproject/PRJNA552984>

[Team, Rstudio. 2015. “RStudio: Integrated Development for R. Boston, MA: RStudio.” Inc.](http://paperpile.com/b/qiUfmY/rC1yi)

Testis Assembly. Kinsella et al. 2019. [Programmed DNA Elimination of Germline Development Genes in Songbirds](http://paperpile.com/b/ggxh4E/FHid). Figshare. P7359_106.phased.fasta.gz. <https://doi.org/10.6084/m9.figshare.8852024>

[Wickham, Hadley. 2011. “ggplot2.” *Wiley Interdisciplinary Reviews: Computational Statistics* 3 (2): 180–85.](http://paperpile.com/b/qiUfmY/6DOv6)
