# Supplementary material for: A Transcriptomic Pipeline Adapted for Genomic Sequence Discovery of Germline-Restricted Sequence in Zebra Finch, Taeniopygia guttata
Source: Genome Biol Evol. 2021 Apr 26;13(6):evab088. doi: 10.1093/gbe/evab088 (PMC8245190; doi:10.1093/gbe/evab088)
Supplement: evab088_Supplementary_Data — Supplementary data are available at Genome Biology and Evolution online. [file evab088_supplementary_data.zip › suppTable2.pdf]

**Gene**	**Sequence (5’ to 3’)**
β-actin forward	TGGAGAAGAGCTACGAACTCCCTG
β-actin reverse	GAAAGATGGCTGGAACAGGGCCTC
Splicing factor 38A forward	CACACGCGTATCTACGAATCCAAGTAC
Splicing factor 38A reverse	CTGTCAGTCTCATGTACAGTGCTCC
BMP15 forward	GAGACCTCGCCTTCAGAGCTCAAGG
BMP15 reverse	CTACATGCTGAATCTGTACCGGCGTG
VEGFA forward	GAAGCTTTGGTAAGAGACTGGGAAAC
VEGFA reverse	CATGATTAGCCAGACTATATATTTGGCTAC
DPH6 forward	CCTCTGTGAAGCACTCTAGAGC
DPH6 reverse	CATGTAGGCAGTACAGCCTCTAG
GRC Non-coding sequence forward	CAAACCCACACATCGGTAAATTCAG
GRC Non-coding sequence reverse	GTTGAAGATTTCCATTTACATCAGTGC
GBE1 forward	AGCATAGTCAAAGAGCCTGCTGTC
GBE1 reverse	GAGTCCTGATTATGCCACGTTGTG
Methyltransferase forward	CATCACACACAGTTGTGGCTTGG
Methyltransferase reverse	CAAAGGGATATTCCTGACCACATGAC
SCRIB forward	GGTGAACGGAGTGTCCCTGCAC

SCRIB reverse	GAGGCAGGTGGAGAACCTCTCG
A-Chromosome Non-coding forward	CACTGATTGAAGGAGATGATTGTGTGC
A-Chromosome Non-coding reverse	GACACAGAGCCACAAGCTGCAG
RPL4 forward	GGTTTACGCCTCGCAGCGCATG
RPL4 reverse	CAGAAGAGCGAGCGTGTCCCAG
