# Supplementary material for: A Transcriptomic Pipeline Adapted for Genomic Sequence Discovery of Germline-Restricted Sequence in Zebra Finch, Taeniopygia guttata
Source: Genome Biol Evol. 2021 Apr 26;13(6):evab088. doi: 10.1093/gbe/evab088 (PMC8245190; doi:10.1093/gbe/evab088)
Supplement: evab088_Supplementary_Data — Supplementary data are available at Genome Biology and Evolution online. [file evab088_supplementary_data.zip › suppFig1.pdf]

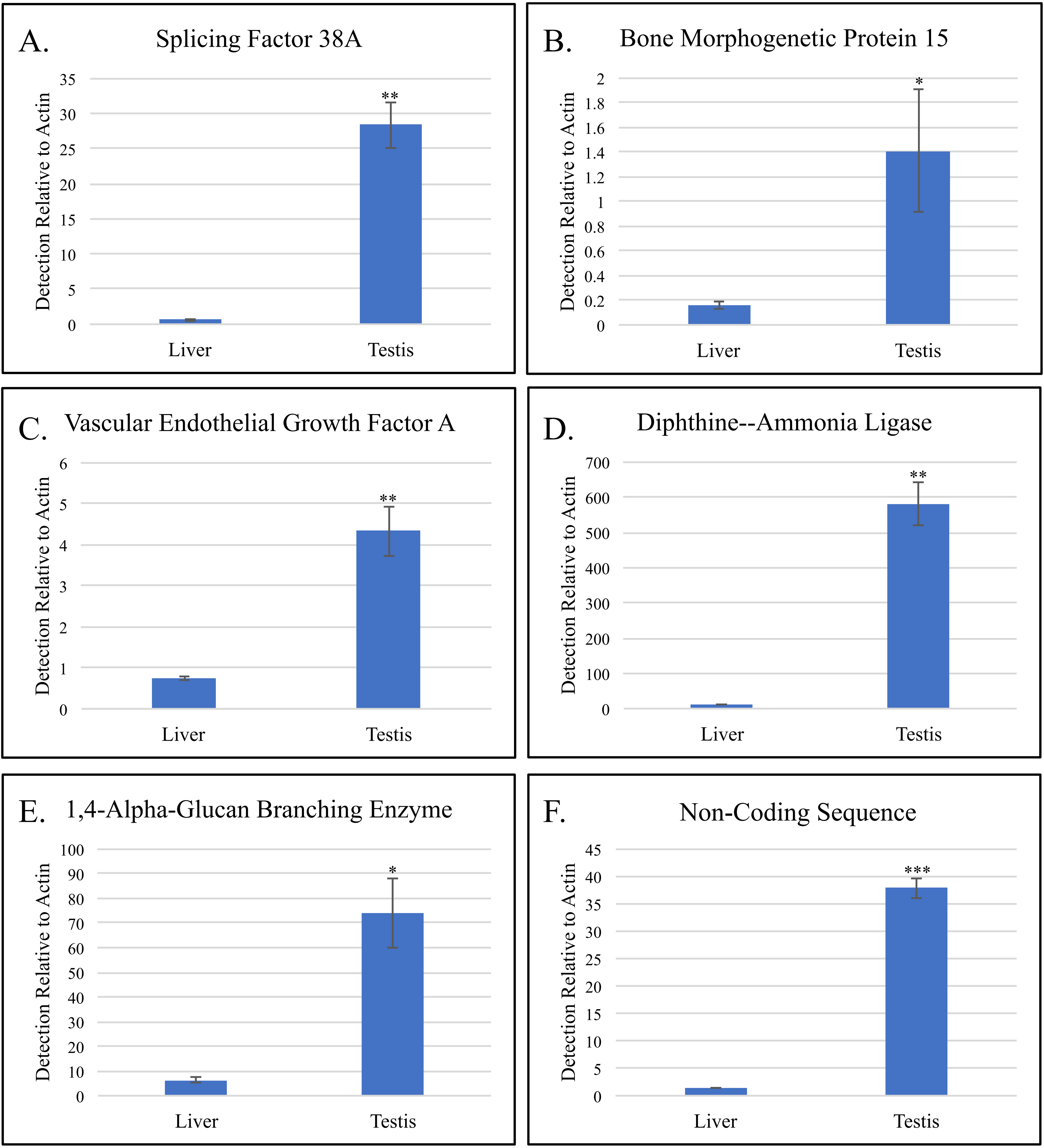

A.
Splicing Factor 38A
Detection Relative to Actin
35
30
25
20
15
10
5
0
**
Liver
Testis
B.
Bone Morphogenetic Protein 15
Detection Relative to Actin
2
1.8
1.6
1.4
1.2
1
0.8
0.6
0.4
0.2
0
*
Liver
Testis
C.
Vascular Endothelial Growth Factor A
Detection Relative to Actin
6
5
4
3
2
1
0
**
Liver
Testis
D.
Diphthine--Ammonia Ligase
Detection Relative to Actin
700
600
500
400
300
200
100
0
**
Liver
Testis
E.
1,4-Alpha-Glucan Branching Enzyme
Detection Relative to Actin
100
90
80
70
60
50
40
30
20
10
0
*
Liver
Testis
F.
Non-Coding Sequence
Detection Relative to Actin
45
40
35
30
25
20
15
10
5
0
***
Liver
Testis
