# Supplementary material for: A Transcriptomic Pipeline Adapted for Genomic Sequence Discovery of Germline-Restricted Sequence in Zebra Finch, Taeniopygia guttata
Source: Genome Biol Evol. 2021 Apr 26;13(6):evab088. doi: 10.1093/gbe/evab088 (PMC8245190; doi:10.1093/gbe/evab088)
Supplement: evab088_Supplementary_Data — Supplementary data are available at Genome Biology and Evolution online. [file evab088_supplementary_data.zip › suppFigure2.pdf]

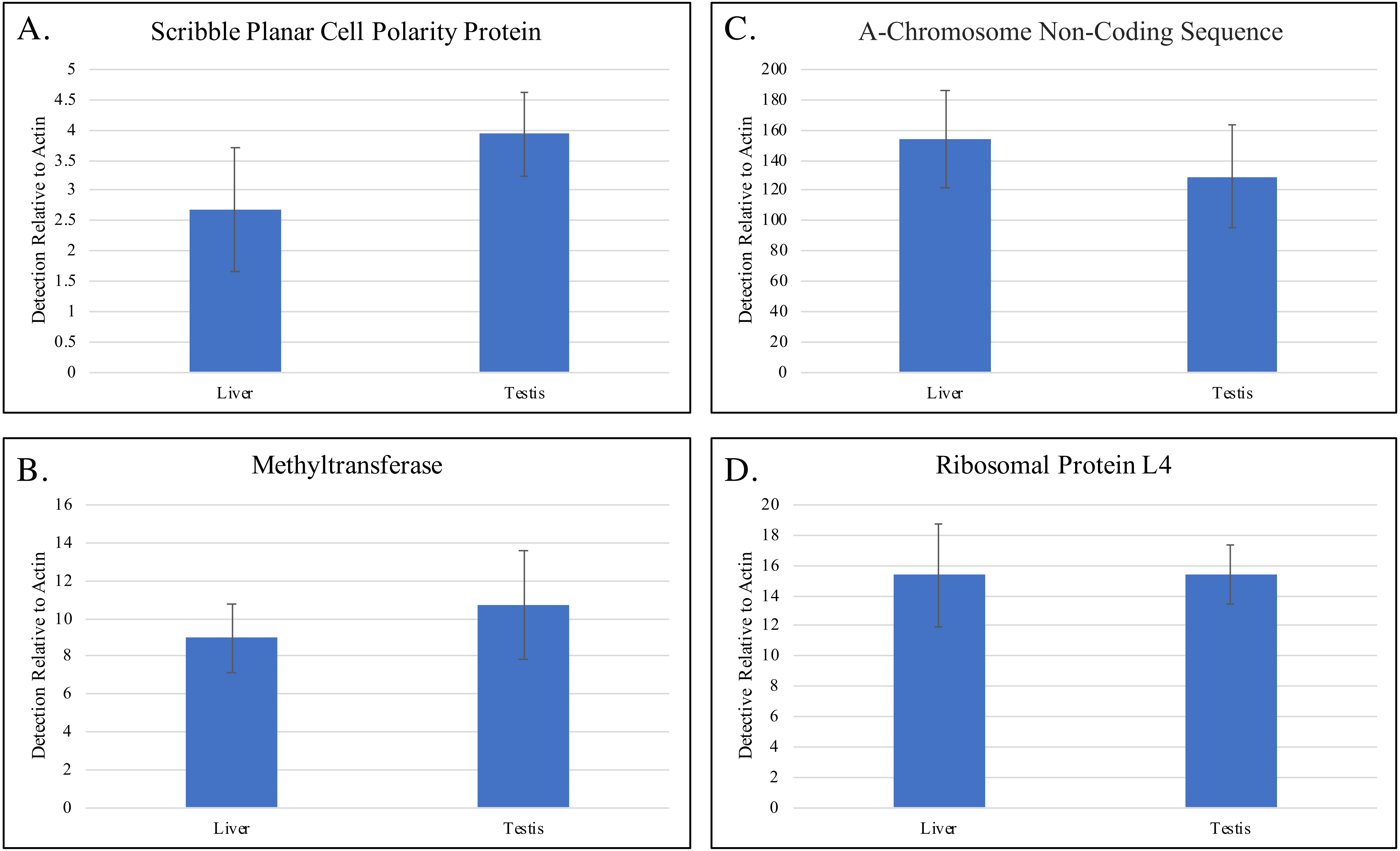

A.
Scribble Planar Cell Polarity Protein
Detection Relative to Actin
0
0.5
1
1.5
2
2.5
3
3.5
4
4.5
5
Liver
Testis
B.
Methyltransferase
Detection Relative to Actin
0
2
4
6
8
10
12
14
16
Liver
Testis
C.
A-Chromosome Non-Coding Sequence
Detection Relative to Actin
0
20
40
60
80
100
120
140
160
180
200
Liver
Testis
D.
Ribosomal Protein L4
Detective Relative to Actin
0
2
4
6
8
10
12
14
16
18
20
Liver
Testis
