# Supplementary figures and images for: A Transcriptomic Pipeline Adapted for Genomic Sequence Discovery of Germline-Restricted Sequence in Zebra Finch, Taeniopygia guttata
Source: Genome Biol Evol. 2021 Apr 26;13(6):evab088. doi: 10.1093/gbe/evab088 (PMC8245190; doi:10.1093/gbe/evab088)

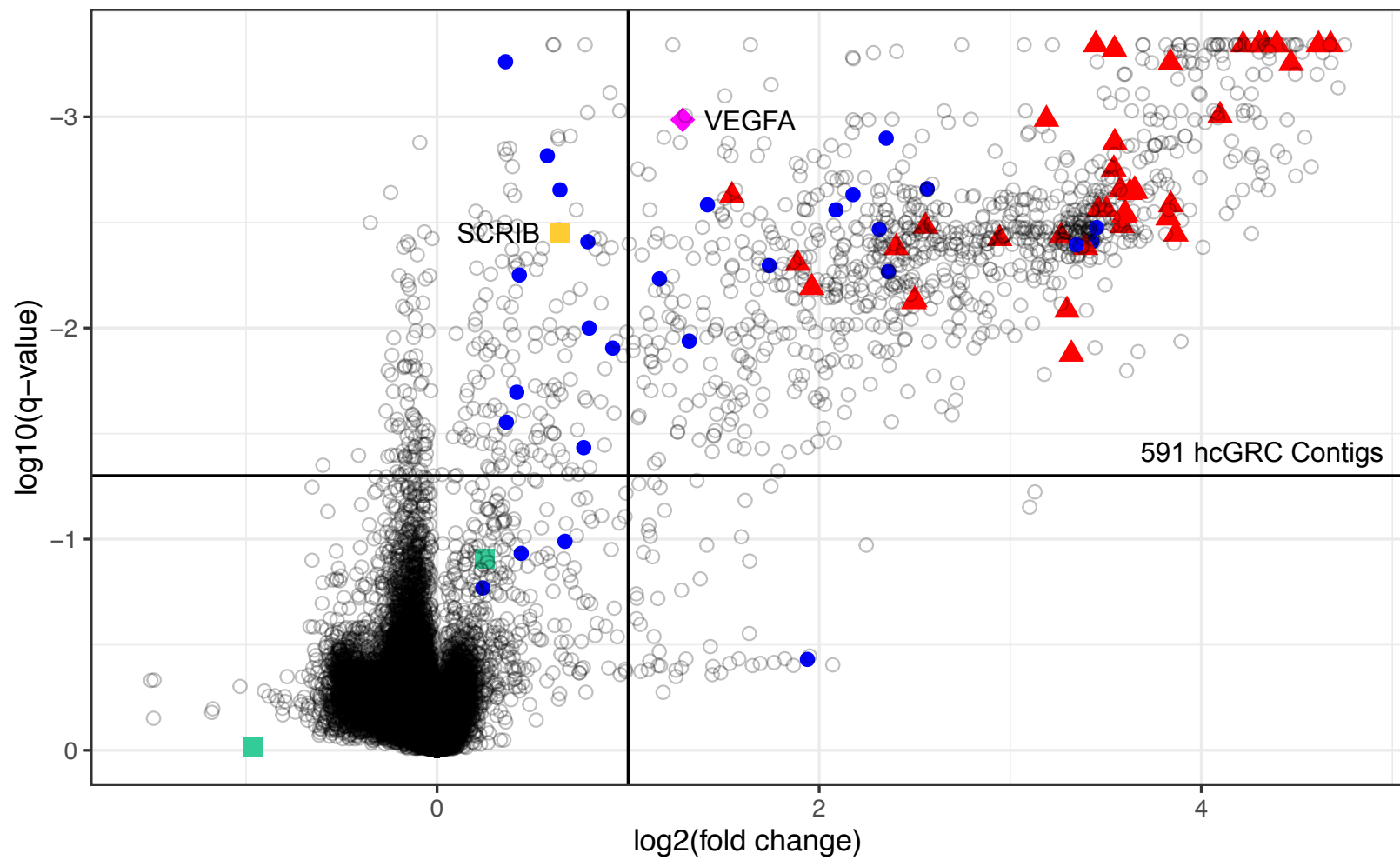

Supplement: evab088_Supplementary_Data — Supplementary data are available at Genome Biology and Evolution online. [file evab088_supplementary_data.zip › suppFig3.pdf]
